# Non-therapeutic administration of a model antimicrobial growth promoter modulates intestinal immune responses

**DOI:** 10.1186/1757-4749-3-14

**Published:** 2011-09-25

**Authors:** Estela Costa, Richard RE Uwiera, John P Kastelic, L Brent Selinger, G Douglas Inglis

**Affiliations:** 1 Zoonotic Bacteriology, Lethbridge Research Centre, Lethbridge, Alberta, Canada; 2 Department of Biological Sciences, University of Lethbridge, Lethbridge, Alberta, Canada; 3 Department of Agricultural, Food and Nutritional Science, University of Alberta, Edmonton, Alberta, Canada

**Keywords:** Antimicrobial growth promoters, AGP, chlortetracycline, Citrobacter rodentium, immunomodulation hypothesis

## Abstract

**Background:**

The development of efficacious alternatives to antimicrobial growth promoters (AGP) in livestock production is an urgent issue, but is hampered by a lack of knowledge regarding the mode of action of AGP. The belief that AGP modulate the intestinal microbiota has become prominent in the literature; however, there is a lack of experimental evidence to support this hypothesis. Using a chlortetracycline-murine-*Citrobacter rodentium *model, the ability of AGP to modulate the intestinal immune system in mammals was investigated.

**Results:**

*C. rodentium *was transformed with the tetracycline resistance gene, *tet*O, and continuous oral administration of a non-therapeutic dose of chlortetracycline to mice did not affect densities of *C. rodentium *CFU in feces throughout the experiment or associated with mucosal surfaces in the colon (i.e. at peak and late infection). However, chlortetracycline regulated transcription levels of Th1 and Th17 inflammatory cytokines in a temporal manner in *C. rodentium*-inoculated mice, and ameliorated weight loss associated with infection. In mice inoculated with *C. rodentium*, those that received chlortetracycline had less pathologic changes in the distal colon than mice not administered CTC (i.e. relative to untreated mice). Furthermore, chlortetracycline administration at a non-therapeutic dose did not impart either prominent or consistent effects on the colonic microbiota.

**Conclusion:**

Data support the hypothesis that AGP function by modulating the intestinal immune system in mammals. This finding may facilitate the development of biorationale-based and efficacious alternatives to AGP.

## Background

The in-feed administration of non-therapeutic doses of antimicrobial growth promoters (AGP) has been successfully used to promote animal growth for more than 60 years [1]. Unfortunately, the indiscriminate use of AGP [2] has contributed to the emergence of antimicrobial resistance (AMR) in zoonotic pathogens [3-5], and the European Union (EU) implemented a precautionary ban on administration of all AGP (i.e. at non-therapeutic doses) to livestock [6,7]. The AGP ban in the EU increased the therapeutic administration of antimicrobial agents [6], as well as the cost of animal production, and resulted in a general decline in livestock production [8]. A recent guidance document issued by the United States Food and Drug Administration recommended restrictions that would limit the use of AGP [9]. Thus, it is anticipated that an AGP ban will progressively be imposed in North America, adversely affecting the sustainability of livestock production. We contend that elucidating the mode of action of AGP will facilitate the development of suitable, efficacious, and biorationale-based alternatives to AGP to enhance livestock production.

The literature on the mode of action of AGP is scarce; the most widely accepted hypothesis is that AGP modulate the intestinal microbiota [10,11]. The 'microbiota modulation hypothesis' suggests that AGP reduces microbial competition for nutrients, decreases production of growth-depressing metabolites by intestinal microorganisms, suppresses opportunistic pathogens, and results in a thinner intestinal wall, which increases nutrient assimilation [10]. Despite widespread acceptance, definitive evidence to support the microbiota modulation hypothesis is lacking. The consistency of growth-promotion effects imparted by AGP on various animal species possessing highly dissimilar intestinal microbiota coupled with low concentrations at which AGP are administered (i.e. at doses less than the minimum inhibitory concentration for most pathogens) challenges the validity of the microbiota modulation hypothesis of AGP action [12]. Since many antimicrobial agents have anti-inflammatory and immunomodulatory properties [13], an alternate hypothesis for the mode of action of AGP was recently proposed [12], namely, that AGP decrease immunologic stress in the host [14]. The intestinal mucosa is in a constant state of "physiologic inflammation" [15], attributable to the close contact between the intestinal mucosa and the microbiota [16]. By decreasing immunologic stress in the intestinal mucosa, AGP would reduce the catabolic cost to the host, thereby increasing the energy available for muscle development and improving growth performance [17]. The 'immunomodulation hypothesis' for AGP action is consistent with the growth-promotion effects observed when AGP is administered to animals possessing very disparate intestinal microbiota [12]. To our knowledge, the validity of the immunomodulation hypothesis for AGP action has not been formally tested. Thus, we formulated and tested the hypothesis that non-therapeutic concentrations of a model AGP administered orally to mammals will modulate enteric immune responses.

To test the immunomodulation hypothesis of AGP action we used chlortetracycline (CTC) as a model AGP; CTC is a commonly used AGP in North American livestock production, and at therapeutic concentrations, tetracyclines are known to modulate immune responses [18]. Given the inherent genetic and microbial variability of livestock species, mice were used as a mammalian model. To test the immunomodulation hypothesis it was necessary to experimentally induce inflammation within the intestine of mice, and to do so, we utilized *Citrobacter rodentium*. This bacterium is a non-invasive, attaching/effacing bacterial pathogen which causes a self-limiting acute colitis in immunocompetent laboratory mice [19,20], and has been widely accepted as a robust *in vivo *model system to assess host-pathogen interactions [20,21]. *C. rodentium *colonizes the apical surface of the large intestinal mucosa, causing mucosal hyperplasia, localized microvilli loss, and mucosal inflammation [22]. Using the CTC-murine-*C. rodentium *model, specific experimental objectives were to: determine the temporal impact of CTC on host responses in mice inoculated or not inoculated with *C. rodentium*; and concomitantly measure the effects of CTC and/or *C. rodentium *on the composition of the mucosa-associated colonic microbiota.

## Results

### Residual CTC in feces

Residual CTC was detected in acidic extracts of feces from mice given CTC, with no significant difference between the CTC and CTC+CR treatments. Mean residual CTC value was 63.9 ± 6.6 μg/g of feces for CTC treatment mice, and 63.3 ± 9.6 μg/g of feces for CTC+CR treatment mice. No CTC was detected in acidic extracts from feces of mice not given CTC.

### Intestinal colonization by *C. rodentium *and *tet*O temporal occurrence

**Figure 1:**
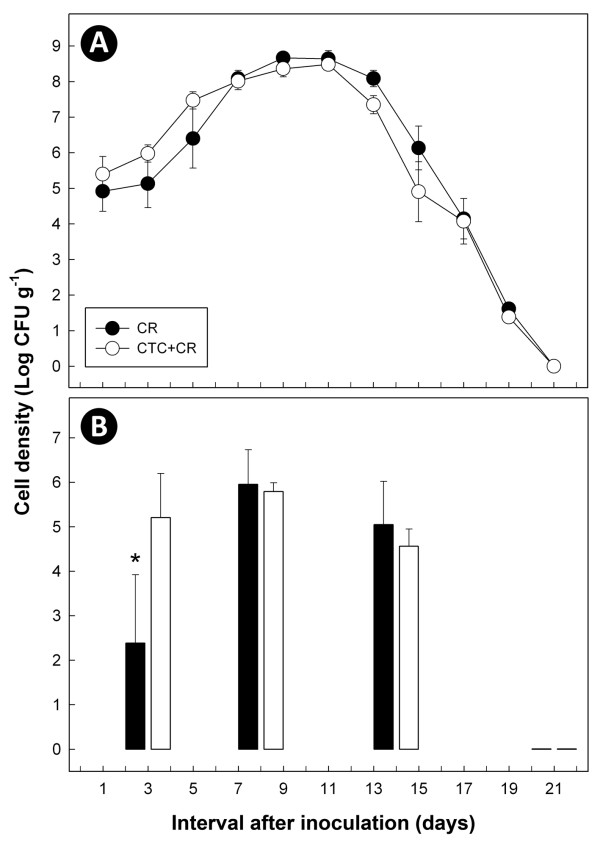
**Mean quantities of *C. rodentium *cells (CFU/g) in feces (A) and associated with colonic mucosa (B) of mice inoculated with the bacterium and given CTC (CTC+CR) or water alone (CR)**. Vertical lines associated with individual markers at each sample time indicate standard error of the mean (n = 3). *Values differ (P < 0.05).

Considerable numbers of *C. rodentium *cells were recovered from feces and colonic mucosa of inoculated mice throughout the experiment (Figure 1). No *C. rodentium *cells were recovered from uninoculated mice. Densities of *C. rodentium *cells recovered from feces and mucosal surfaces changed over time (P < 0.001); population sizes peaked between 9 and 11 days p.i., and decreased thereafter until no cells were detected 21 days p.i. (Figure 1). Relative to the CR treatment, administration of CTC did not affect (P = 0.81) densities of *C. rodentium *CFU in murine feces (P = 0.84) at all sample times (Figure 1A). With the exception of the 3 day p.i. sample (P < 0.05), there also was no difference (P > 0.05) between treatments in densities of *C. rodentium *CFU associated with colonic mucosa (Figure 1B).

All *C. rodentium *cells recovered and evaluated from the feces of CTC+CR treatment mice had the *tet*O gene until day 19 p.i. All *C. rodentium *cells recovered and evaluated from CR treatment mice had the *tet*O gene until day 9 p.i.; 50% of cells had the *tet*O gene at days 11 and 13 p.i., and no cells possessed the *tet*O gene at days 15, 17, 19, and 21 p.i.

### Signs of disease

**Figure 2:**
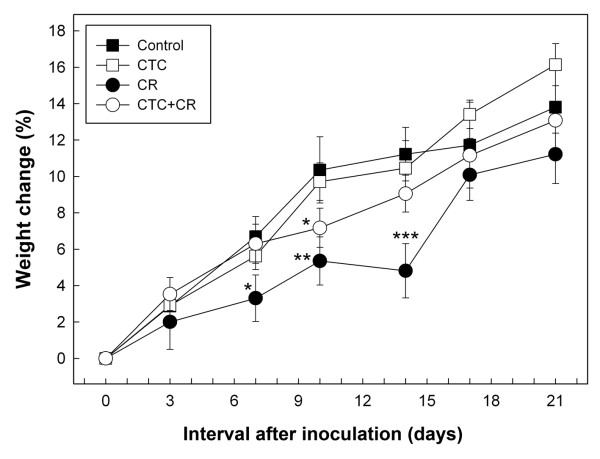
**Mean change in body weight (%) of mice**. Treatments are: (Control) mice not inoculated with *C. rodentium *or administered CTC; (CTC) mice not inoculated with *C. rodentium *but administered CTC; (CR) mice inoculated with *C. rodentium *but not administered CTC; and (CTC+CR) mice inoculated with *C. rodentium *and administered CTC. Vertical lines associated with individual markers at each sample time indicate standard error of the mean (n = 3). *P < 0.05 when compared with Control mice; **P < 0.05 when compared with Control or CTC treatment mice; ***P < 0.05 when compared with Control, CTC or CTC+CR treatment mice.

Mice inoculated with *C. rodentium *(i.e. CR treatment and CTC+CR treatment) did not exhibit clinical signs of disease. However, CR treatment mice weighed less (P < 0.05) than Control treatment mice on days 7, 10, and 14 p.i. (Figure 2). Mice inoculated with *C. rodentium *and given CTC (i.e. CTC+CR treatment) weighed less (P < 0.05) than Control mice only on day 10 p.i., and weighed more (P < 0.05) than CR treatment mice on day 14 p.i. There was no difference (P > 0.05) in body weight between Control and CTC treatment mice. However, an apparent trend of CTC treated mice being heavier than Control animals was observed on day 17 and 21.

### Gross and histopathology

**Figure 3:**
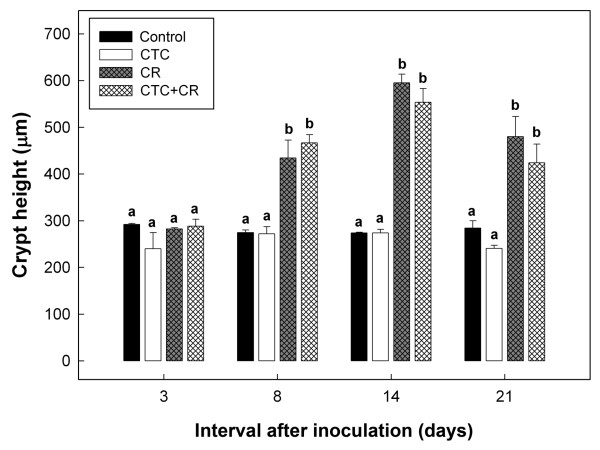
**Mean colonic epithelial crypt height (μm)**. Treatments are: (Control) mice not inoculated with *C. rodentium *or administered CTC; (CTC) mice not inoculated with *C. rodentium *but administered CTC; (CR) mice inoculated with *C. rodentium *but not administered CTC; and (CTC+CR) mice inoculated with *C. rodentium *and administered CTC. Vertical lines associated with histogram bars indicate standard error of the mean (n = 3). Histogram bars at individual times not indicated with the same letter differ (P < 0.05).

Thickening of the distal colon was observed in four of the six CR and CTC+CR treatment mice at day 8 p.i. Conspicuous thickening of the distal colon was evident 14 days p.i. in all CR treatment mice and approximately two-thirds of CTC+CR treatment mice. All *C. rodentium*-inoculated mice (i.e. CR and CTC+CR treatments) had colonic pathology (detected histologically) relative to Control mice (Tables 1-2). The intensity of inflammation increased throughout the infection period with the highest scores of inflammation observed at 14 days p.i. Notably, transmural inflammation was observed in the colons of CR treatment mice at day 14 p.i. Infection by *C. rodentium *increased (P < 0.05) crypt height, which was first evident 8 days p.i., and crypt height remained significantly different relative to Control and CTC treatment mice throughout the remainder of the experimental period (Figure 3). Crypt height reached a maximum 14 days p.i. (P < 0.05, Figure 3). Crypt height was not different (P > 0.05) between CTC+CR and CR treatments, nor between Control and CTC treatments. Total pathology scores and individual category scores representing tissue changes did not differ (P > 0.05) between treatments at day 3 p.i. (Table 2). At subsequent sample times, there was no difference (P > 0.05) between Control and CTC treatments, nor between CTC+CR and CR treatments. At day 8 p.i., total score and all individual category scores differed (P < 0.05) for CTC+CR and CR treatments relative to the Control treatment. Although there was no significant difference between CTC+CR and CR treatments, there was a trend for decreased pathologic changes in CTC+CR treatment at later stages of infection. For example, there was no difference (P > 0.05) in mitotic activity scores for the CTC+CR relative to the Control treatment at 14 days p.i., whereas mitotic activity was higher (P < 0.05) for the CR treatment relative to the Control treatment (Table 2). At day 21 p.i., crypt height, inflammatory infiltrates, and total pathology scores differed (P < 0.05) between the Control and CR treatments, but did not differ (P > 0.05) between the Control and CTC+CR treatments.

**Table 1:** Histopathological scores of the distal colons of mice

	Control				CTC				CR				CTC+CR			
				
Cat/Day	3	8	14	21	3	8	14	21	3	8	14	21	3	8	14	21
EH^a^	0^b^	0	0	0-1	0-1	0-1	0-1	0	0-1	1-2	2-4	1-2	0	2	1-3	0-1
CH	0	0	0	0	0-1	0	0-1	0	0-1	2	2-4	1-2	0-1	2-3	1-3	0-2
EI	0	0	0	0	0-1	0	0	0	0	3-4	2-3	0-2	0	3	0-4	0-2
II	0	0-1	0	0	0	0	0	0	0	2-4	3-4	1-4	0	3-4	1-4	0-2
MA	0	0-1	0	0	0	0	0	0	0	2-3	3	1	0-1	2-3	1-3	1-2
GC	0	0	0	0	0	0	0	0	0	1-2	2-3	0-1	0	1-2	0-3	0
Total	0	0-1	0	0-1	0-2	0-2	0-2	0-1	0-1	13-15	16-20	4-12	0-1	14-16	4-20	1-9

^a^Pathology categories (Cat): EH, epithelial hyperplasia; CH, crypt height; EI, epithelial injury; II, inflammatory infiltrates; MA, mitotic activity; GC, globlet cell depletion. Total score corresponds to the sum of scores of all categories.

^b^Range of scores were: 0 to 4 for EH, CH, EI, II; 0 to 3 for MA, GC; 0 to 22 for Total.

**Table 2:** Pairwise analyses of histopathological changes in the distal colons of mice^a^

	Control vs CR				Control vs CTC+CR				CR vs CTC+CR			
			
Cat/Day	3	8	14	21	3	8	14	21	3	8	14	21
EH^b^	0.317	0.034*	0.076	0.090	1.000	0.025*	0.075	0.456	0.317	0.317	0.261	0.197
CH	0.317	0.025*	0.076	0.034*	0.317	0.039*	0.068	0.114	1.000	0.317	0.486	0.796
II	1.000	0.046*	0.068	0.037*	1.000	0.043*	0.068	0.121	1.000	0.637	0.814	0.261
EI	1.000	0.034*	0.068	0.114	1.000	0.025*	0.197	0.317	1.000	0.317	0.816	0.456
MA	1.000	0.043*	0.045*	0.025*	0.317	0.043*	0.068	0.034*	0.317	0.456	0.317	0.317
GC	1.000	0.034*	0.068	0.317	1.000	0.034*	0.182	1.000	1.000	1.000	0.796	0.317
Total	0.114	0.046*	0.037*	0.046*	0.114	0.046*	0.037*	0.072	1.000	0.261	1.000	0.513

^a^Pairwise comparisons were conducted using the Kruskal Wallis non-parametric test. P values marked with an asterisk are significantly different (P = 0.05).

^b^Pathology categories (Cat): EH, epithelial hyperplasia; CH, crypt height; EI, epithelial injury; II, inflammatory infiltrates; MA, mitotic activity; GC, globlet cell depletion. Total score corresponds to the sum of scores of all categories.

### Cytokine gene expression

**Figure 4:**
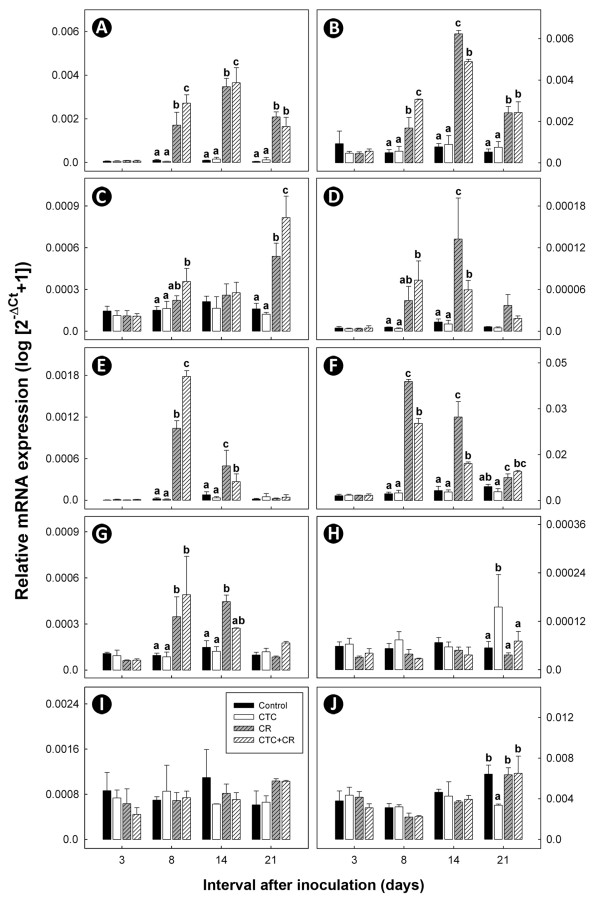
**Relative mean mRNA expression (log [2^-ΔCt ^+ 1]) of cytokine genes in colonic tissues**. Treatments are: (Control) mice not inoculated with *C. rodentium *or administered CTC; (CTC) mice not inoculated with *C. rodentium *but administered CTC; (CR) mice inoculated with *C. rodentium *but not administered CTC; and (CTC+CR) mice inoculated with *C. rodentium *and administered CTC. Vertical lines associated with histogram bars indicated standard error of the means (n = 3). (A) IFN-γ; (B) TNF-α; (C) IL-2; (D) IL-17A; (E) IL-22; (F) IL-1β; (G) IL-6; (H) IL-4; (I) IL-10; (J) TGF-β. For each cytokine, histogram bars at individual times indicated by a different letter differ (P < 0.05).

Concentrations of Th1- (IFN-γ, TNF-α, and IL-2), Th2- (IL-4), Th17- (IL-17A, IL-22, IL-1β, and IL-6), and Treg- (IL-10 and TGF-β1) cytokine mRNA in colonic tissues was quantified. *C. rodentium *infection increased (P < 0.05) concentrations of Th1 cytokine mRNA (Figure 4A-B) and Th17 cytokine mRNA (Figure 4D-G) at 8 and 14 days p.i. Transcript levels of IL-2 were differentially elevated (P < 0.05) in inoculated mice at 21 days p.i. only (Figure 4C). However, no significant changes in transcript levels of genes associated with Th2 (Figure 4H) and Treg (Figure 4I-J) cytokines were detected in inoculated mice. Mice from the CTC treatment did not show differential mRNA expression for most cytokines relative to the Control treatment. However, there was a two fold decrease (P < 0.05) in the concentration of TGF-β mRNA (Figure 4J) and a near two fold increase (P < 0.05) in IL-4 mRNA (Figure 4H) in CTC treatment mice on day 21 p.i. CTC also increased (P < 0.05) transcript levels of IL-2 (Figure 4C).

In inoculated mice, CTC administration (i.e. CTC+CR treatment) increased (P < 0.05) transcript levels of IFN-γ, TNF-α, and IL-22 genes (Figure 4A-B, Figure 5E), and decreased (P < 0.05) transcript levels of the IL-1β gene 8 days p.i. relative to the CR treatment (Figure 4F). Furthermore, administration of CTC to inoculated mice increased (P < 0.05) transcript levels of the IFN-γ, and decreased (P < 0.05) transcript levels of the TNF-α, IL-17A, IL-22, and IL-1β genes 14 days p.i. relative to the CR treatment (Figure 4A-B, Figure 4D-F). For Th17 cytokines, there was a trend for an initial increase in transcript levels of IL-17A, IL-22, and IL-6 genes in infected mice administered CTC (i.e. CTC+CR treatment) relative to CR treatment mice (i.e. at day 8 p.i.) (Figure 4D-E, Figure 4G). Thereafter (i.e. at day 14 p.i.), transcript levels of IL-17A, IL-22, and IL-6 cytokine genes tended to decrease differentially in CTC+CR treatment mice relative to CR treatment mice. Transcript levels of IL-1β in CTC+CR mice remained lower relative to CR treatment mice at both the peak and late infection periods (Figure 4F).

### Mucosa-associated bacterial communities

Diverse bacterial communities were observed in association with mucosal surfaces of the colons of all mice, regardless of treatment. The composition of bacterial communities varied amongst individual animals; however, ordination of T-RFLP profiles by NMS analysis revealed no obvious grouping of replicate mice within treatments (not shown). Global comparisons revealed the formation of unique (P < 0.05) community compositions for the Control and CTC+CR treatments at 8 days p.i., and for the CTC+CR and CR treatments at 21 days p.i. (Table 3). The composition of the colonic microbiota in mice infected by *C. rodentium *and/or given CTC differed (P < 0.05) from mice assigned to corresponding control treatments, but this occurred periodically, and only at early and peak infection (Table 4). Furthermore, differences were not consistent for both treatments (i.e. for individual pairwise comparisons) and this was attributed to variability amongst replicate animals. Based on NMS analysis, the microbiota of mice administered CTC clustered only at day 3 p.i. (not shown).

**Table 3:** Global analyses of T-RFLP community profiles^a^

	Treatment			
	
Day	Control	CTC	CR	CTC+CR
3	0.888	0.249	0.487	0.069
8	0.003*	0.708	0.287	0.033*
14	0.391	0.709	0.575	0.717
21	0.070	0.085	0.035*	0.035*

^a^P values marked with an asterisk are significantly different (P ≤ 0.05).

**Table 4:** Pairwise analyses of T-RFLP community profiles^a^

	Day / Group							
	3		8		14		21	
				
Treatment Comparisons	A	B	A	B	A	B	A	B
Control (A) vs CTC (B)	0.797	0.021*	0.025*	0.732	0.338	0.542	0.308	0.267
Control (A) vs CR (B)	0.908	0.423	0.023*	0.441	0.570	0.547	0.181	0.151
Control (A) vs CTC+CR (B)	0.807	0.024*	0.053	0.153	0.298	0.599	0.059	0.063
CTC (A) vs CR (B)	0.193	0.633	0.508	0.269	0.808	0.691	0.058	0.065
CTC (A) vs CTC+CR (B)	0.827	0.593	0.738	0.127	0.698	0.746	0.212	0.218
CR (A) vs CTC+CR (B)	0.416	0.042*	0.227	0.008*	0.450	0.653	0.057	0.052

^a^P values marked with an asterisk are significantly different (P = 0.05).

## Discussion

Research regarding the use of AGP in agriculture and animal production has predominantly focused on measuring the prevalence of AMR in zoonotic pathogens [4,5,23-25]. That giving AGP selects for resistance to antimicrobials in bacteria has promulgated the belief that AGP induce growth promotion in livestock as a result of direct effects on the intestinal microbiota [10,26]. However, there is a dearth of experimental evidence to support the 'microbiota modulation hypothesis'. Furthermore, it was recently reported that AGP administration did not significantly alter the intestinal microbiota in pigs [27]. Antibiotics are anti-inflammatory and immunomodulatory [13]; thus, AGP may function by modulating the immune system within the intestine thereby providing a catabolic advantage to the host [12]. To our knowledge this has never been tested. We formulated the 'immunomodulation hypothesis' of AGP action, and we used a CTC-murine-*C. rodentium *model to test this hypothesis.

An antimicrobial agent is a growth promoter when administered in/on the feed of food animals to promote growth and enhance feed efficiency. Growth promoters are usually administered in relatively low concentrations (i.e. non-therapeutic), ranging from 2.5 to 125 mg kg^-1 ^(ppm), depending on the compound and animal species treated [28]. CTC is commonly used as an AGP for mammalian livestock [29]. In the current study, a simulated non-therapeutic dose of CTC was given to mice with the dose extrapolated from that commonly used in beef cattle. Measurable quantities of ingested CTC (and its epimer, epi-CTC) are excreted in livestock feces [30]. Consistent with this finding, measurable CTC concentrations were excreted in the feces of all mice given CTC. The experimental design required that mice be continuously administered CTC (i.e. to mice assigned to the CTC and CTC+CR treatments), necessitating that the *C. rodentium *be transformed to have resistance to CTC. Although tetracycline resistance determinants may persist in the absence of selection pressure [31,32], the plasmid-borne *tet*O gene transferred into *C. rodentium *was less persistent in the mice not given CTC. However, there was no significant difference between the CR and CTC+CR treatments in shedding or mucosal colonization by the bacterium at the peak, late, and clearance stages of infection. Reasons for the significantly lower numbers of *C. rodentium *associated with colonic mucosa in mice not administered CTC at day 3 p.i. is unknown, but is likely due to sampling variability as there was no difference between the treatments in densities of *C. rodentium *cells shed in feces at day 3 p.i. or any other sample time.

We utilized mice that were reared under conditions in which intestinal inflammation would be expected to be exceptionally low (i.e. in the absence of inducers of immune responses such as *C. rodentium*), and as such, differential immunomodulation between the control and CTC treatment would be expected to be minimal. However, we observed that the expression of the cytokine genes IL-4 and TGF-β were markedly different in CTC mice as compared to control animals on 21 day p.i. The increase in IL-4 was both statistically and biologically significant (i.e. two times greater than in control animals). Importantly, both CR infected and uninfected mice that received CTC presented with modest to markedly significant higher concentrations of IL-4 at clearance relative to Control mice, thus suggesting that CTC contributes to the differentiation of Th2 cells. Treatment with therapeutic concentrations of tetracycline has been observed to induce changes in IL-4 during both acute and chronic disease by others. For example, Chirgwin et al [33] reported that IL-4 expression was decreased in tetracycline-treated Mongolian gerbils infected with filarial nematodes. In contrast, IL-4 levels in people infected with *Orientia tsutsugamushi *(incitant of scrub typhus) and treated with tetracycline did not change relative to background levels [34]. Although therapeutic concentrations of tetracycline were used in these two studies, their study highlighted the varied responses that tetracycline treatment can have on Th2 cytokine expression within intestinal mucosa. The greater than 50% decrease in TGF-β levels that we observed in CTC mice relative to control mice (statistically and biologically significant) also indicates that the intestine of CTC mice reduces numbers of Treg lymphocytes. The observed effects of CTC on IL-4 and TGF-β are consistent with the immunomodulation hypothesis of AGP action, a conclusion that is consistent with the increased weight gain observed (i.e. a biological trend).

*C. rodentium *was chosen as an activator of inflammation and immunological function because it colonizes the colon of mice and incites acute, self-limited colitis, associated with progressive crypt hyperplasia in the distal colon [19,35]. Consistent with previous reports, there were characteristic pathologic changes in mice inoculated with *C. rodentium*, including thickening of the distal colon wall and crypt elongation attributed to mucosal hyperplasia [36]. We measured changes in intestinal morphology as opposed to weighing colons as hypertrophy of the muscularis externa, deposition of submucosal collagen (fibrosis), and infiltration of large numbers of inflammatory cells can all cause marked increases in colonic weight that are independent to changes in the mucosa. In addition to hyperplasia, we observed multifocal transmural inflammation and infiltration of inflammatory cells to the submucosa, muscularis externa, and serosa, as documented previously [22,37]. *C. rodentium *induces strong Th1 and Th17 cell-mediated immune responses [36,38-40], and there were significant increases in transcript levels of both Th1 (INF-γ, TNF-α, IL-2) and Th17 (IL-17A, IL-22, IL-1β, IL-6) cytokines in mice infected with the bacterium. These effector T helper cell responses are important in preventing and eliminating enteric bacterial infections. Th17 cells have a crucial role in the clearance of extracellular bacterial pathogens that are not adequately handled by Th1 or Th2 responses [38,41], and Th1 cells are needed to eliminate prolonged infections [42]. Moreover, morphological changes in the intestine, namely increased enterocyte proliferation and turnover, were associated with increased expression of the Th17 cytokines, IL-17 [43] and IL-22 [44], an observation consistent with the present study. One of the key observations of this study was the time dependant activation of Th1 and Th17 of T helper cells. Based on differential expression of Th1 and Th17 cytokine profiles, we inferred there was a temporal relationship in cytokine expression necessary for eliminating infection by enteric pathogens, such as *C. rodentium*. The initial increase in Th17 cytokines may be needed to induce an effective immune response, but could be inadequate to completely clear an infection; therefore, subsequent induction of Th1 cytokines may be required to clear prolonged infections. This trend has been observed in other enteric pathogen and animal models. As an example, mice infected with *Salmonella enterica serovar Typhimurium* had a marked Th17 and Th1 response in the early and late stages of infection, respectively [42].

Consistent with the immunomodulation hypothesis, administration of a non-therapeutic dose of CTC decreased *C. rodentium*-induced weight loss and mitigated pathologic changes, albeit in a modest manner. For example, at 14 day p.i., mitotic activity was significantly increased for the CR relative to the Control treatment, but not for the CTC+CR relative to the Control treatment. Similarly, crypt height, inflammatory infiltrates, and the cumulative score were significantly increased for the CR treatment, but not for the CTC+CR treatment relative to the Control. Although the reductions in weight loss and histopathologic changes were relatively modest in CTC+CR relative to CR mice, it is apparent that CTC treatment altered immune function as the changes observed in the intestine cannot be explained by CR infection alone. Collectively, these observations are consistent with the immunomodulation hypothesis. We also observed that the administration of CTC (i.e. CTC+CR treatment) altered the expression of cytokine genes involved in the acute phase of inflammation relative to the CR treatment. Concentrations of Th1 cytokines were differentially expressed; IFN-γ mRNA were increased for the CTC+CR treatment at the peak (i.e. day 8 p.i.) and late (i.e. day 14 p.i.) stages of infection, whereas IL-2 mRNA was increased at clearance (i.e. day 21 p.i.), and TNF-α mRNA was increased at the peak stage and decreased at the late stage of infection in mice administered CTC. Concentrations of Th17 cytokines also were differentially expressed between the CTC+CR and CR treatments; IL-17A mRNA was decreased at day 14 p.i., IL-22 mRNA was increased at day 8 p.i. and decreased at day 14 p.i., and IL-1β mRNA was decreased at both days 8 and 14 p.i. An acute-phase response can be triggered by an infectious challenge; it is often characterized by release of the pro-inflammatory cytokines, IL-1β, IL-6, and TNF-α, which orchestrate behavioral, cellular, and metabolic adjustments in the host that alter the partitioning of nutrients away from growth and toward processes that support the immune and inflammatory responses [14]. Furthermore, IFN-γ, IL-1β and IL-6 facilitate TNF-α-induced muscle cachexia [45]. Interestingly, CTC administration only decreased expression of IL-17A mRNA at the late stage of infection in the current study, perhaps due to the concomitant down-regulation of TNF-α and IL-1β mRNA expression. Since the Th17 cell response was amplified by TNF-α and IL-1β [46], it is therefore likely that a decrease in TNF-α and IL-1β expression will also decrease activation of Th17 cells. Taken together, we conclude that reduced pathological changes at the late stage of infection, as well as at clearance, and the absence of weight loss at late infection in inoculated mice that received CTC were due to up-regulation of IL-22 transcripts (i.e. a cytokine important in mucosal defense) at the peak stage of infection, and down-regulation of TNF-α, IL-1β, and IL-17A transcripts (i.e. cytokines involved in inflammation, innate and adaptive immunity) at the late stage of infection, relative to inoculated mice not given CTC.

Our observations that a non-therapeutic concentration of CTC given to mice modulated *C. rodentium*-induced cytokine mRNA expression, ameliorated *C. rodentium*-induced weight loss, and mitigated pathologic changes associated with infection by *C. rodentium*, provided experimental evidence in support of immunomodulation as a primary mechanism of action of AGP in mammalian livestock. There is additional evidence to support the immunomodulation hypothesis. For example, oral administration of tetracycline reduced mortality rates and inflammatory lesions associated with lipopolysaccharide (LPS)-induced septic shock in mice by decreasing serum concentrations of TNF-α [47]. Furthermore, tetracycline inhibited LPS-induced secretion of TNF-α and IL-1β by human monocytes *in vitro *[47]. Similarly, non-therapeutic administration of tetracycline to experimentally stressed poultry reduced immunologic stress by decreasing plasma IL-1 concentrations [17]. Also consistent with the immunomodulation hypothesis, Akunda et al. [48] reported that a low concentration of CTC decreased TNF-α secretion by cultured porcine Kupffer cells that were inoculated with LPS. In addition, intra-peritoneal injection of low doses of tetracycline and doxycycline decreased serum concentrations of IL-1α and TNF-α in mice [49].

The generally accepted microbiota modulation hypothesis of AGP action postulates that AGP modify the microbiota, thereby reducing the deleterious impacts of enteric bacteria on the host [12]. To measure impacts of CTC on the mucosa-associated microbiota of the distal colon, T-RFLP analysis was used. The T-RFLP method is a medium to high resolution method that is commonly used to compare bacterial communities in mammalian intestines [50,51]. The composition of the intestinal microbiota in association with mucosal surfaces can be profoundly affected by inflammatory responses, and sequence-based analysis (i.e. examining the partial 16S rRNA gene) of mucosa-associated and luminal bacteria in mice infected with *C. rodentium *revealed alterations to the microbiota [52]. In contrast, we observed that *C. rodentium *infection did not impart a profound effect on the mucosa-associated microbiota of the murine distal colon in the current study. Reasons for the discrepancies between our study and that of Hoffmann et al. [52] are speculative, but may be because they restricted their examination to communities in the cecum and proximal colon, and communities were only resolved at the phylum or order level by pyrosequencing. Furthermore, they did not measure inter-animal variability. Although pyrosequencing of communities has become prominent in recent years, this platform is not a panacea; sequence reads are currently limited to less than 450 bp thereby limiting relevant sequence data to a small number of variable regions of the 16S rRNA gene, which greatly reduces the ability to discriminate amongst taxa, particularly those that are closely related (e.g. at the species level). One of the major advantages of T-RFLP is its ability to comparatively monitor treatment effects on the microbiota in empirical models by obtaining measures of inter-animal variability. It is possible that the inter-animal variability in the current study obscured subtle effects of *C. rodentium *infection on the colonic microbiota. Alternatively, *C. rodentium *may not exert substantive impacts on the microbiota of the distal colon, since the bacterium does not colonize this region of the colon throughout the entire infection period [53].

Although CTC was given at a non-therapeutic concentration in the current study, it affected the composition of the microbiota, albeit inconsistently and in a non-pronounced fashion. It has been reported that administration of non-therapeutic CTC substantially affected the composition of mucosa-associated microbiota in the ilea of piglets [54]. In contrast, it was reported in a recent study that administration of virginiamycin and tylosin to intensively raised swine at the grow/finishing phase had no effect on bacterial communities in feces [27]. Furthermore, there was a high prevalence of bacteria that carried genes encoding resistance to macrolide-lincosamide-streptogramin B (MLSB), from which they attributed to the negligible impact of AGP on the intestinal community [27]. We have similarly observed that CTC and sulfamethazine, an AGP commonly administered to beef cattle, had no affect on the luminal and mucosa-associated microbiota of the distal small intestine and large intestine of beef cattle (unpublished).

It is evident that both host genetics and environmental factors can affect the intestinal microbiota. For example, persons with gene mutations responsible for the autoinflammatory disorder, familial Mediterranean fever, possessed significantly different bacterial community structures than healthy individuals, even when patients were in remission [55]. Also, Altered Schaedler Flora (ASF) mice of the same genetic background exhibited significant variation in relative cell densities of the eight ASF strains throughout the intestine when mice were maintained in separate cages [56]. The inter-animal variability encountered in the current and previous studies [54,56,57] further illustrates how difficult it is to quantify the impact of a single variable in such a complex system. Although CTC administration imparted a modest effect on the composition of the microbiota in the current study, it was not possible to definitively conclude whether this effect was direct or indirect. Considering a direct effect of CTC on the microbiota, it is unclear how and to what degree subtle changes in the microbiota caused by CTC would induce growth promotion, especially given the complexity of the mechanisms involved and that AGP exert an effect in animals with highly dissimilar microbiota. Since the indigenous microbiota can influence *C. rodentium *pathogenesis [22], even subtle changes in the microbiota may have influenced immune responses to *C. rodentium*, further illustrating the complexity of the interaction and the difficulty of elucidating specific effects.

## Conclusions

The mucosa-associated microbiota of the murine colon was not dramatically affected by *C. rodentium*, and was inconsistently affected by oral administration of non-therapeutic CTC. Furthermore, non-therapeutic CTC administration modulated immune responses that were temporally related to *C. rodentium *infection, in accordance with the immunomodulation hypothesis of AGP action. Although it was not possible to definitively ascertain whether non-therapeutically administered CTC directly or indirectly modulated the murine immune system, we inferred that modulation of the microbiota alone was not responsible. The current study not only broadens our knowledge of how AGP may exert an effect, it also emphasizes the necessity of examining host responses interactively with the intestinal microbiota to elucidate the mechanisms of action of AGP. Furthermore, an altered focus on strategies that modulate the enteric immune system as opposed to those that modify the microbiota may lead to the development of efficacious alternatives to AGP. Additional research is required to further define the mechanisms of immunomodulation exerted by CTC and other AGP. In this regard, studies utilizing gnotobotic animals in concert with cell culture models are warranted, and such studies have been initiated by our research group.

## Methods

### Inflammation incitant

*C. rodentium *(ATCC 51459) was transformed with a gene that confers resistance to tetracycline. Plasmid pMEK91, a *Campylobacter *shuttle vector that carries the *tet*O gene [58], was modified (EcoRI digestion and re-ligation) to remove the green fluorescent protein gene. This vector was transferred into *C. rodentium *by electroporation as previously described [59]. Electroporated cells were incubated for 1 hour at 37°C and 100 rpm, plated on Luria-Bertani (LB) agar dishes containing 50 μg/mL tetracycline hydrochloride (Sigma, St. Louis, MO), and incubated overnight at 37°C. To confirm resistance to tetracycline in the transformed strain, a minimum inhibitory concentration test was performed as previously described for tetracycline hydrochloride [5]; *Escherichia coli *(ATCC 25922) was used as the quality control strain.

### Mice and treatments

Forty eight female, 4-week-old C57BL/6J specific pathogen free (SPF) mice were purchased from Charles River Laboratories International, Inc. (Montreal, QC). Mice were housed at the small animal facility located at Agriculture and Agri-Food Canada Lethbridge Research Centre (AAFC LRC). All requirements specified by the Canadian Council on Animal Care were met, and the project was approved by the LRC Animal Care Committee before commencement (Animal Use Protocol Review 0915). Mice were housed in sterilized, filter-topped cages throughout the experiment, and maintained on a 12 hour light/dark cycle. Mice were randomly assigned to one of the following treatments: (i) no CTC, no inoculation (Control); (ii) 32 mg/L CTC, no inoculation (CTC); (iii) no CTC, *C. rodentium *inoculation (CR); and (iv) 32 mg/L CTC, *C. rodentium *inoculation (CTC+CR). The dose of CTC used in mice was extrapolated from non-therapeutic doses administered to cattle. The ratio of the non-therapeutic (75 mg/day) to therapeutic (6000 mg/day) doses of CTC in cattle is 1:80. By dividing the average therapeutic dose in mice (i.e. 640 mg/L) by 80, an estimated non-therapeutic dose for mice is 8 mg/L. Since the average therapeutic dose in mice is approximately four fold higher than in cattle, the 8 mg/L dose was multiplied by four to account for metabolic differences between the two animals, and thus a non-therapeutic dose of 32 mg/L of CTC was administered to mice. Administration of CTC to mice commenced 3 weeks before inoculation with *C. rodentium*, and continued throughout the experimental period. CTC was added to autoclaved drinking water, and fresh solutions were provided twice weekly. Control and CR treatment mice were provided with autoclaved water alone. Sucrose (5% w/v; Sigma, St. Louis, MO) was added to all water solutions to enhance palatability, and water bottles were covered with aluminum foil.

### Inoculation and maintenance of mice

To prepare inoculum, recombinant *C. rodentium *cells were cultured in LB broth containing 50 μg/mL of tetracycline for 16 hours at 37°C and 100 rpm, centrifuged for 5 minutes at 1600 × g, the supernatant removed, and the pelleted cells were resuspended in sterile phosphate buffer saline (PBS; 10 mM sodium phosphate buffer, 130 mM sodium chloride; pH 7.2) to a final concentration of 10^9 ^CFU/mL. Sodium bicarbonate (2% w/v) was added to the inoculum. Mice (CR and CR+CTC treatments) were inoculated with 100 μL (10^8 ^CFU) of the inoculum by oral gavage on two consecutive days. Mice not inoculated with *C. rodentium *(Control and CTC treatments) were orally gavaged with an equal volume of PBS containing 2% sodium bicarbonate on the same days. Mice were provided with food and water *ad libitum*, and were weighed twice weekly. In addition, mice were monitored for signs of disease according to the Animal Care Committee criteria for stress assessment.

### Collection of feces and tissues

Feces were collected aseptically from all mice once a week for estimating residual CTC, and every second day (commencing 1 day after the initial gavage) for enumeration of *C. rodentium*. Mice were humanely euthanized by anesthesia with isofluorane (Halocarbon Products Corporation, River Edge, NJ) followed by cervical dislocation 3, 8, 14, and 21 days post-inoculation (p.i.), corresponding to the early, peak, late infection, and clearance periods, respectively [20]. Twelve mice were euthanized at each time point (i.e. three mice per treatment; n = 48 total). After euthanasia, a midline incision was made, and the entire colon was rapidly harvested and examined for alterations in macroscopic appearance [36]. Four 1-cm sections from the colon were then collected distally to cranially for histology, quantification of cytokine mRNA expression, characterization of mucosa-associated microbiota, and *C. rodentium *enumeration, respectively. Colon samples collected for histology were preserved in 10% buffered formalin. Samples collected for RNA extraction were inserted in sterile microcentrifuge tubes containing RNAlater (Qiagen Inc., Mississauga, ON) and kept at -20°C until processed; tissues were placed in RNAlater within 2-3 min after death. To characterize the microbiota, the colonic section was opened longitudinally, the mucosal surface gently rinsed with chilled sterile PBS taking care not disrupt mucus, tissue samples were aseptically removed with a sterile 3-mm-diameter Biopsy Acu-Punch (CDMV, St. Hyacinthe, QC), and samples were kept at -20°C until processed. Colonic samples collected for enumeration of *C. rodentium *were placed on ice immediately after collection, and processed within 2 hours.

### Estimation of residual CTC in murine feces

To estimate the concentration of residual CTC in fecal samples, the basic agar diffusion method described previously was used [60]. Briefly, residual CTC was extracted by suspending known amounts of feces (10-50 mg) in 0.01 N HCl. Feces were homogenized, the homogenate centrifuged (12,000 × g, 20 minutes), the supernatant recovered (i.e. acidic extract) and sterilized by filtration through a 0.2 μm filter (Nalgene, Rochester, NY), and 50 μL of the sterile extract was added to individual wells established with a cork borer (4-mm-diam) in Tryptic Soy Agar (TSA; BD, Franklin Lakes, NJ) in Petri dishes to which cells of *Staphylococcus aureus *strain ATCC 29213 had been distributed over the agar surface. *S. aureus *cells were obtained from an overnight culture on TSA at 37°C, cells were suspended in Columbia broth (BD), cell density adjusted to an optical density (OD) equivalent to a 0.5 McFarland standard (OD of 0.1 at 600 nm), and 100 μL aliquot of the cell suspension spread on TSA. Assays were carried out in triplicate for each sample. Zones of inhibition in the *S. aureus *lawn were measured after 18 hours at 37°C, using a Biomic V3 Image Analyzer (Giles Scientific USA, Santa Barbara, CA), with slight adjustments performed by hand. Estimation of CTC concentrations in fecal samples was interpolated from a standard curve obtained by measuring zones of inhibition from known concentrations of acidified CTC.

### Enumeration of *C. rodentium*

Densities of *C. rodentium *cells were determined by homogenizing feces or colonic samples in Columbia broth, and spreading serial dilutions of the homogenate onto MacConkey agar (BD). Cultures were incubated overnight at 37°C, enumerated at the dilution yielding 30-300 CFU/dish, and adjusted by weight. *C. rodentium *colonies were identified based on morphology [61], and representative colonies exhibiting characteristic morphology (i.e. an average of three colonies per dish) were subcultured to confirm their identity and the presence of the *tet*O gene by PCR. Reaction mixtures for PCR consisted of 2 μL of a suspension of cells in 20 μL of Optima water, 1X PCR buffer, 0.2 mM of each deoxynucleoside triphosphate, 0.1 μg/μL of acetylated bovine serum albumin (Promega, Madison, WI), 0.625 Units of Taq DNA polymerase (Qiagen Inc.), 0.5 μM of each primer, and Optima water to a final volume of 20 μL. For *C. rodentium *identification, the Cr-espB-f (5'-AAGTCTGTCAATACCGCCTC-3') and Cr-espB-r (5'-AATGTGCCAACTGTCTCATC-3') primers were used [62]. For *tet*O gene detection, the tetO-F-PstI (5'-TAA CTG CAG AGA TTC AGT ATT ATA ACA AGG-3'), and tetO-R-PstI (5'-TTA CTG CAG CAT CAT AAT TAT CTC TAA TCC-3') primers were used [58].

### Histopathology

Tissue samples were maintained in 10% buffered formalin for a minimum of 4 hours and for a maximum of 2 weeks. Tissue samples were dehydrated with ethanol and Histoclear (Fisher Scientific Inc.), and paraffinized with Paraplast Plus (Fisher Scientific Inc.) for 2 hours at 60°C in a vacuum oven. Samples were embedded using a Shandon Histocentre III (Fisher Scientific Inc.), sectioned (4 μm) using a Finesse 325 microtome (Fisher Scientific Inc.), and sections were placed on Superfrost Plus Gold slides (Fisher Scientific Inc.). Sections were deparaffinized with xylene, stained with hematoxylin and eosin (H&E) following a standard protocol, and examined with a Zeiss Axioskop III (Carl Zeiss Canada Ltd., Toronto, ON). Images were captured using an Axiocam camera (Carl Zeiss Canada Ltd.). Histological inflammation scoring was performed in a "blinded" fashion (i.e. as to treatment) by a veterinary pathologist, with scoring criteria adapted from previously described methods [19,63]. Colonic sections were graded 0 to 4 for epithelial cell hyperplasia, crypt height, epithelial injury, extent of inflammatory infiltrates, and 0 to 3 for mitotic activity of epithelial cells, and goblet cell depletion. The total pathology score was obtained by calculating the sum of scores for all categories for each mouse. Epithelial hyperplasia caused by *C. rodentium *(i.e. crypt height) was quantified by calculating the average of ten measurements of well-oriented crypts for each section.

### Quantification of mRNA expression of cytokine genes

Total RNA was extracted from colonic tissues with TRIzol reagent (Invitrogen Corp., Carlsbad, CA) according to the manufacturer's recommendations. Residual genomic DNA contamination was removed using a DNA purification protocol from RNeasy kit (Qiagen Inc.). After quantification on an Ultrospect 3100 pro UV/Visible spectrophotometer (General Electric Healthcare, Piscataway, NJ), 1 μg of total RNA was reverse transcribed into cDNA using the RT2 First Strand Kit (SABiosciences Corp., Frederick, MD). Quality assurance and control of total RNA was performed with the RT2 RNA QC PCR Arrays Kit. A custom RT2 Profiler PCR Array System (SABiosciences Corp.) was used to quantify the mRNA expression of the following cytokines: interleukin (IL)-1β, IL-2, tumor necrosis factor (TNF)-α, interferon (IFN)-γ, IL-6, IL-4, IL-10, IL-17A, IL-22, and transforming growth factor (TGF)-β1. Two housekeeping genes, glyceraldehyde 3-phosphate dehydrogenase (GAPDH) and actin-β were used to provide an estimate of the range of threshold cycles to be expected in subsequent PCR array analyses. The housekeeping gene that presented the least variation of expression among samples with the quality control kit was used to normalize the data. Real-time PCR was performed using an MxPro 3005 thermocycler (Agilent Technologies, Santa Clara, CA). Expression was normalized against GAPDH, and data were log-transformed (i.e. log [2^-ΔCt ^+ 1]) for statistical analysis.

### Characterization of bacterial communities

Total genomic DNA was extracted from colonic samples using the Gram-positive bacteria protocol of the DNeasy Blood and Tissue Kit (Qiagen Inc.). Amplification of the 16S rRNA genes for terminal restriction fragment length polymorphism (T-RFLP) analysis, restriction of amplified rRNA genes, capillary gel electrophoresis, and fragment size determination, were conducted as performed as previously described [51]. Selection of terminal restriction fragments (T-RFs) (i.e. "true peaks") was performed with T-REX software [64].

### Statistical analyses

The experiment was designed as a randomized complete block design (2 × 2 factorial) with: (i) two levels of treatment (i.e. CTC or no CTC, and *C. rodentium *or no *C. rodentium*); (ii) four (i.e. for *C. rodentium *CFU associated with colonic mucosa, cytokine mRNA transcription, and colonic crypt height), six (i.e. for murine body weights), or eleven (i.e. for *C. rodentium *CFU in murine feces) levels of time; and (iii) three levels of block as the three replicates were conducted on separate occasions and thus were independent. All analyses were conducted using SAS (SAS Institute Inc., Cary, NC). For parametric data (i.e. CFU counts in feces and colon, murine weight, and cytokine mRNA expression), analysis of variance was performed using the MIXED procedure with treatment one (i.e. CTC or no CTC), treatment two (i.e. *C. rodentium *or no *C. rodentium*), time, and their interaction, included in the model as fixed effects. Differences among means of interest were compared through the generation of least-square means with Fisher's protected least significant difference test. For *C. rodentium *CFU counts in feces and murine body weights, the repeated-measurement statement was applied, and the appropriate error structure was determined using Akaike's information criterion and the Bayesian information criterion. In all instances, the UNIVARIATE procedure was used to produce normal probability plots to confirm normality and to identify outliers, which were removed before completion of the final analysis. For non-parametric data (i.e. histological inflammation scoring data for individual category scores and total scores), the NPAR1WAY procedure with the Wilcoxon scores (rank sums) for variable score and one-sided Wilcoxon two-sample test were performed. For analysis of T-RFLP data, T-RF matrices produced by the T-REX software were imported into Bionumerics (Applied Maths, Inc., Austin, TX) for cluster analyses and group significance determination as described previously [51]. Cluster analysis was performed using the Dice coefficient, and non-metric multi-dimensional scaling analysis (NMS) of the similarity matrices was performed using SAS (SAS Institute Inc.). For all analyses, P < 0.05 was considered significant.

## Competing interests

The authors declare that they have no competing interests.

## Authors' contributions

EC participated in the design of the study, performed experiments, conducted data analyses, and co-drafted the manuscript. RREU participated in the design of the study, conducted histopathological evaluations, co-drafted the pathological and immunological aspects of the manuscript, and edited the manuscript. JPK participated in the design of the study, and edited the manuscript. LBS participated in the design of the study, and served as the academic supervisor for EC. GDI conceived the project, participated in the design of the study, coordinated and supervised the research, assisted with necropsies and tissue collection, co-drafted and edited the manuscript, and co-supervised EC. All authors have read and approved the final manuscript.
